# Preliminary clinical experience with robotic retroperitoneoscopic pancreatic surgery

**DOI:** 10.1186/s12957-018-1468-5

**Published:** 2018-08-16

**Authors:** Guodong Zhao, Zizheng Wang, Minggen Hu, Sai Chou, Xin Ma, Xiangjun Lv, Zhiming Zhao, Yong Xu, Zhipeng Zhou, Rong Liu

**Affiliations:** 1 Military Institution of Hepatopancreatobiliary Surgery, Second Department of Hepatopancreatobiliary Surgery, Chinese People’s Liberation Army (PLA) General Hospital, 28 Fuxing Road, Beijing, 100853 China; 2 Department of Urology, Chinese People’s Liberation Army (PLA) General Hospital, 28 Fuxing Road, Beijing, 100853 China

**Keywords:** Pancreas, Neoplasm, Distal pancreatectomy, Enucleation, Robotic surgery, Retroperitoneoscopic surgery

## Abstract

**Backgrounds:**

Retroperitoneoscopic surgery has shown advantages in urological surgery. However, its application in pancreatic surgery for neoplasm is rare. Robotic surgical system with its magnified view and flexible instruments may provide a superior alternative to conventional laparoscopic system in retroperitoneoscopic surgery. We aimed to evaluate the safety, feasibility, and short-term outcomes in a series of patients treated by robotic retroperitoneoscopic pancreatic surgery.

**Case presentation:**

Between March 2016 and May 2016, four patients with solitary pancreatic neuroendocrine neoplasms were treated with robotic retroperitoneoscopic surgery. Prospective collected clinical data were retrospectively analyzed. Three patients underwent distal pancreatectomy (one combined with resection of left adrenal adenoma), and one patient enucleation. The mean operative time was 80 min (range 30–110 min). The estimated blood loss was insignificant. There was no conversion to open procedure. The mean postoperative hospital stay was 5.25 days (range 4–6 days). The mean tumor size was 1.375 cm (range 1.0–1.8 cm) in diameter. All patients’ blood glucose level returned to normal range within 1 week postoperatively. Two patients had pancreatic biochemical leak. No patients underwent subsequent treatment, and no recurrence occurred during the 12-month follow-up period.

**Conclusions:**

This study preliminarily indicates that robotic retroperitoneoscopic pancreatic surgery is safe and feasible for neoplasms in the dorsal portion of distal pancreas in selected patients, with some potential advantages of straightforward access, simple and fine manipulation, short operative time, and fast recovery.

## Background

For lesions in the distal pancreas, enucleation and distal pancreatectomy are the major treatments in open, laparoscopic, and robotic approaches. However, because of the special anatomical location of the pancreas, the dorsal portion of distal pancreas is difficult to expose through conventional transperitoneal approach and the transperitoneal operation may also interfere the organs in the peritoneal cavity and induce accidental injuries to organs. Inspired by urological retroperitoneoscopic surgery, we performed the first retroperitoneoscopic pancreatic enucleation in 2010 [1] and thereafter performed dozens of cases of retroperitoneoscopic pancreatic surgery (RPS). We find that RPS has numerous potential advantages, including straightforward operative approach, simplified manipulation, and fluent postoperative drainage, which could significantly reduce the incidence of secondary complications related to pancreatic fistula [1]. However, narrow space and confined activity impede the safety and further application of RPS [2]. Compared to conventional laparoscopic and retroperitoneoscopic surgery, robotic surgery offers a clear and steady 3-D vision as well as the flexible and delicate operation with reduced hand tremor. Thus, the advantages of robotic surgery are best represented in the precise surgical operation in narrow space, such as robotic radical prostatectomy, which has already become the “gold standard” practice in many west countries after more than 10 years of implementation [3–5].

Our surgical team has accumulated extensive experience of more than 1800 cases of robotic hepato-biliary-pancreatic surgery [6, 7]. Thereafter, we started the clinical application of robotic retroperitoneoscopic pancreatic surgery (RRPS) and try to explore the safety and feasibility of this modified retroperitoneoscopic pancreatic surgery. The aim of this study was to report our experience and analyze short-term operative outcomes of a cohort of patients who underwent RRPS.

## Case presentation

**Fig. 1:**
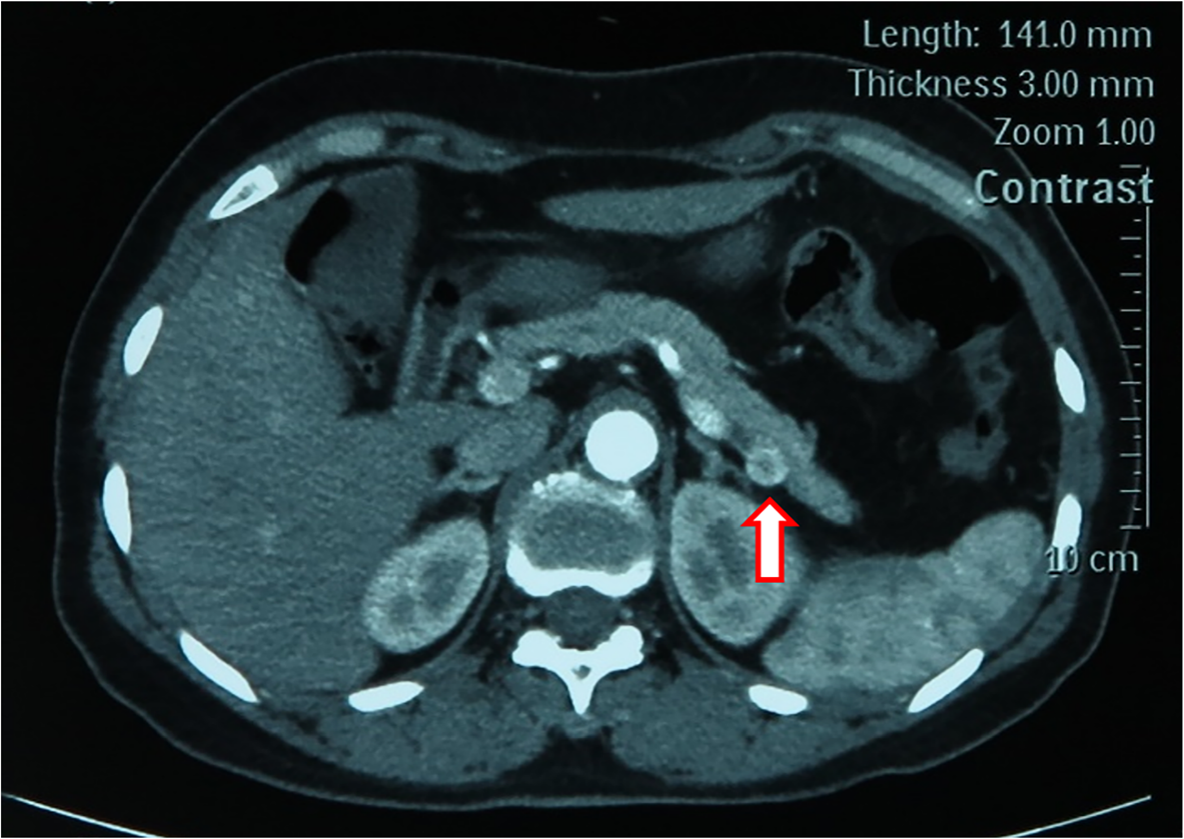
Contrast-enhanced CT showing a tumor located in the dorsal portion of pancreatic tail, close to splenic vessels. The arrow indicates the tumor

Between March 2016 and May 2016, four consecutive patients with solitary pancreatic neuroendocrine neoplasms (pNENs) were treated with robotic retroperitoneoscopic surgery (Da Vinci SI; Intuitive Surgical, Inc., Sunnyvale, CA, USA) at Chinese People’s Liberation Army (PLA) General Hospital, Second department of Hepatopancreaticobiliary Surgery. The demographic data and perioperative outcomes were summarized in Table 1. This series consisted of four female patients with a mean age of 51 years (range 41–58 years). All patients manifested Whipple’s triad (symptoms of hypoglycemia when fasting or during exercise, hypoglycemia measured during onset of symptoms, and symptom relief after glucose administration) for more than 1 year. Two cases had tumor located in the dorsal portion of pancreatic tail (Fig. 1) and two cases in the dorsal portion of distal pancreatic body. The lesions were diagnosed and positioned preoperatively by multimodal imaging, including endoscopic ultrasonography (EUS), CT scanning, magnetic resonance imaging (MRI), and selective PET-CT. Patients’ data were retrospectively collected, including demographic characteristics, clinical information, perioperative outcomes, and 12-month following-up outcomes. Informed consent was obtained from all patients before the operation, and the study was approved by the ethics committee of the hospital and the procedures were in accordance with the Helsinki Declaration.

**Table 1:** Demographic data and perioperative outcomes of patients undergoing robotic retroperitoneoscopic pancreatic surgery

Baseline characteristics					Intraoperative outcomes				Postoperative pathology		Postoperative outcomes	
No.	Sex	Age (years)	BMI (kg/m^2^)	Operation history	Operation name	Operative time^a^ (min)	Blood loss (ml)	Injury of peritoneum	Pathology (grade of pNENs)	Tumor diameter (cm)	Postoperative hospital stays (day)	Pancreatic fistula^b^
1	Female	58	25.6	Hysterectomy	Enucleation	30	–	No	G1	1.5	4	None
2	Female	57	22.2	None	Distal pancreatectomy and resection of adrenal adenoma	110	20	No	G2	1.0	6	Biochemical leak
3	Female	41	36.3	None	Distal pancreatectomy	100	–	Yes	G1	1.8	5	None
4	Female	48	24.4	None	Distal pancreatectomy	80	–	No	G1	1.2	6	Biochemical leak

^a^Manipulative time in the retroperitoneal cavity, without skin incision, closure, retroperitoneal space establishment, and docking time for the robot

^b^Referred to International Pancreatic Fistula Group Criteria (2016)

The operations were performed by Dr. Rong Liu (Console Surgeon) and Dr. Guodong Zhao (Bedside Surgeon). In two cases, the trocar placement and the establishment of artificial retroperitoneal space were assisted by Dr. Xin Ma and Dr. Xiangjun Lv from the Urology Department. All the four operations were completed smoothly, three of which were distal pancreatectomy (one case combined with resection of left adrenal adenoma) and one of which was enucleation.

**Fig. 2:**
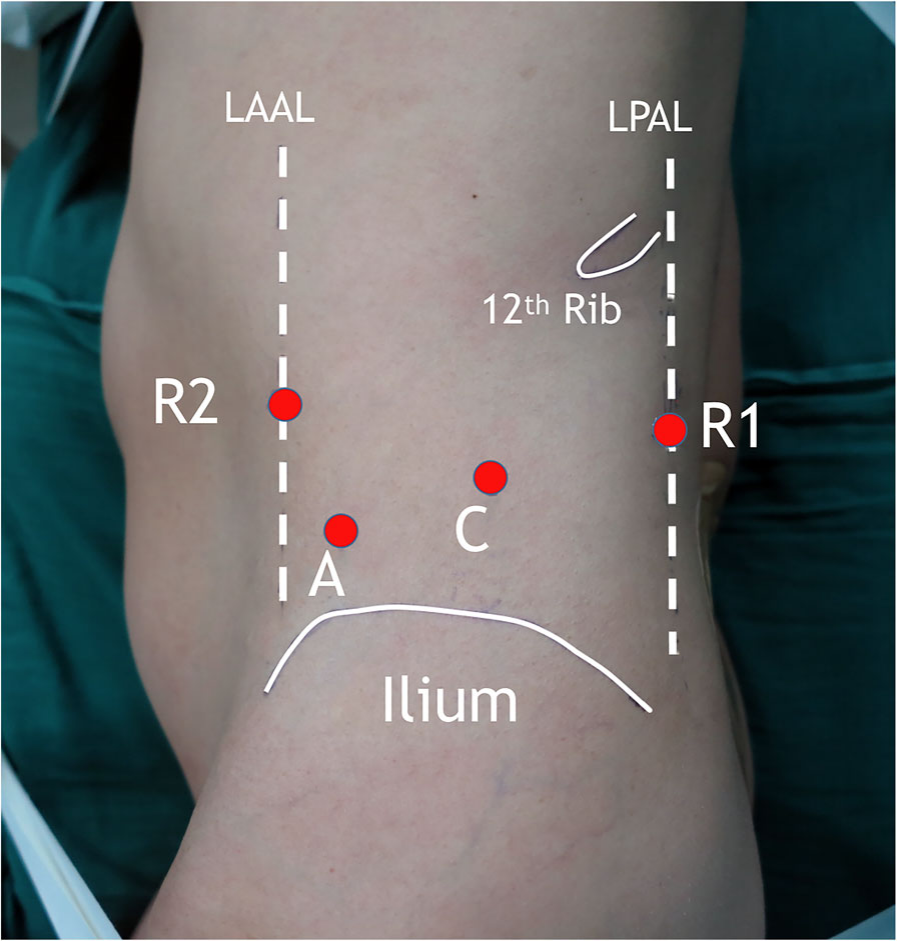
Trocar placement of robotic retroperitoneoscopic pancreatic surgery. LAAL, left anterior axillary line; LPAL, left posterior axillary line; C, camera port; A, assistant port; R1, first robotic arm; R2, second robotic arm

The operative techniques were described as follows: Patients were placed in left lateral decubitus position, and the waist was blocked up to expose the upper flank to the greatest extent [1, 2]. A 2-cm transverse incision was made about 3-cm cephalad to iliac crest. Then, the artificial retroperitoneal space was established using a disposable balloon dilator. Four ports were utilized. The port for the first robotic arm (R1) was placed below the 12th rib at the left posterior axillary line. The port for the second robotic arm (R2) was placed under the 11th rib at the level of the port for the first robotic arm. The camera port (C) was placed to form an obtuse angel or in line with the former two ports. The assistant port (A) was placed lower inferior to the camera port (Fig. 2).

**Fig. 3:**
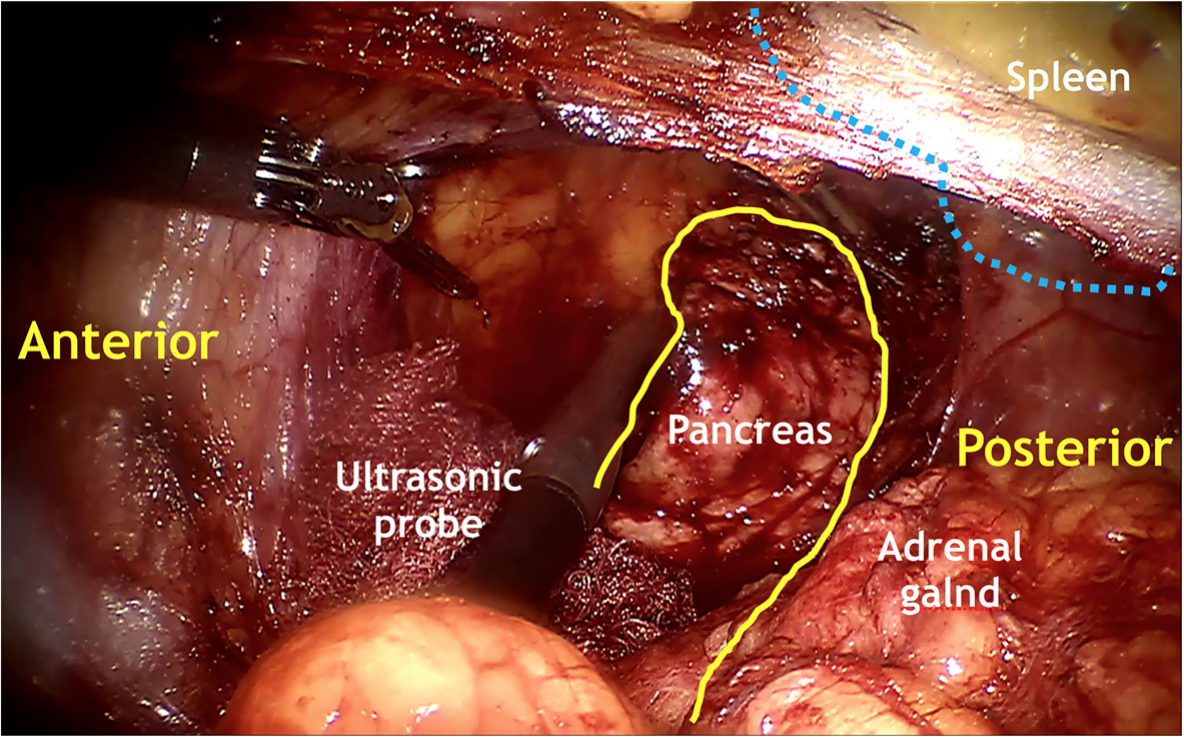
Application of intraoperative ultrasonography to location of the tumor and ensure adequate resection extent after exposing the distal pancreas. Yellow lines, distal pancreas; blue dashed lines, spleen

**Fig. 4:**
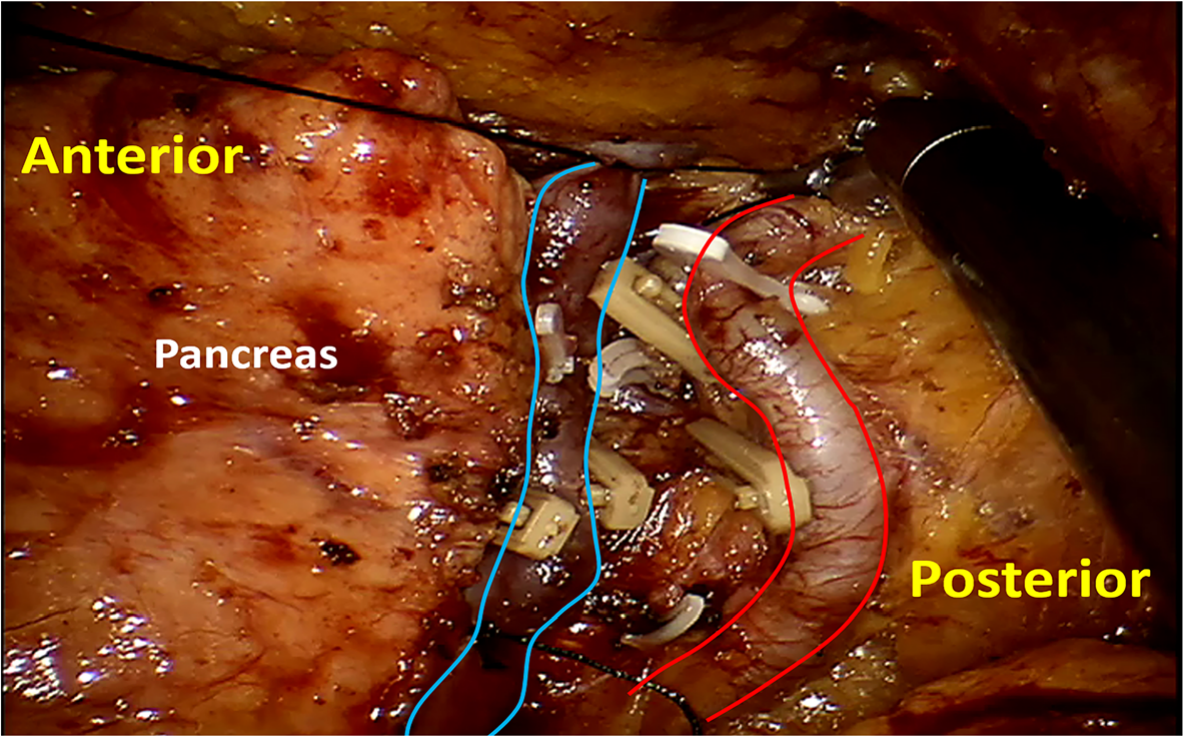
Segmental ligation of splenic vessels before the distal pancreas resection in the robotic retroperitoneoscopy. Blue lines, splenic vein; red lines, splenic artery

**Fig. 5:**
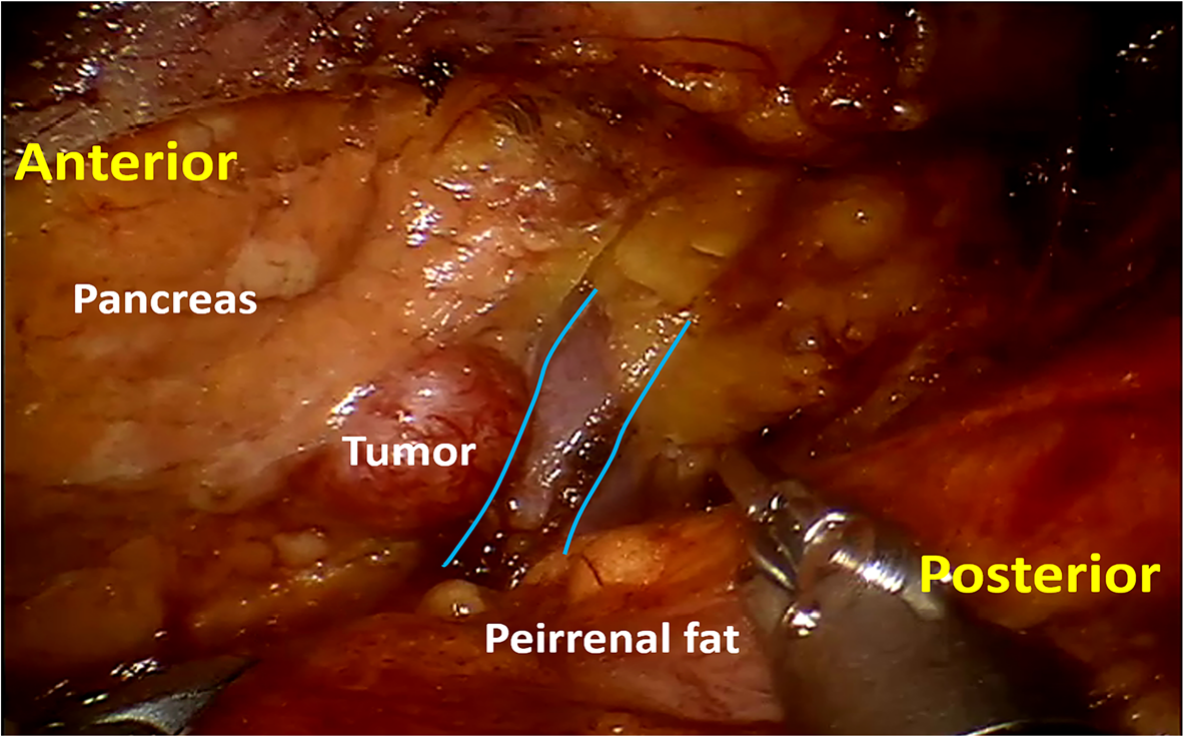
Exposure of the dorsal portion of distal pancreas and location of the tumor in a robotic retroperitoneoscopic pancreatic enucleation. Blue lines, splenic vein; red lines, splenic artery

Firstly, a 0° laparoscope was used. The pararenal fat tissue was dissected using ultrasonic scalpel and retracted inferiorly to expose the posterior renal fascia and lateroconal fascia. The perirenal fascia was then opened lateral to the peritoneal fold. Thereafter, the 0° laparoscope was changed to 30° laparoscope. The perirenal space was expanded by dissecting the perirenal fat towards the adrenal gland. The anterior renal fascia was incised opposite to the adrenal gland, then the anterior pararenal space was dissected and expanded close adjacent to the pancreatic tail. The splenic artery should be carefully kept off during the dissection of anterior pararenal space. When the distal pancreas was exposed, the laparoscopic ultrasonography was performed to re-evaluate the tumor and identify the resection margin (Fig. 3). According to the location of tumor in relation to the main pancreatic duct and splenic vessels, the operative planning was made. The distal pancreatectomy was performed if the tumor was located in the pancreatic tail or adjacent to the main pancreatic duct, and the pancreatic stump was oversewed using continuous suture with 4–0 Prolene suture (Fig. 4). The pancreatic enucleation was performed if the tumor was located in the superficial layer of the distal pancreatic body. The hemorrhage of pancreatic cutting surface was controlled by bipolar electrocoagulation (Fig. 5). Finally, a drain was placed near the distal pancreas through the assistant port, and the incisions were closed after the specimen was retracted.

The mean operative time was 80 min (range 30–110 min). The estimated blood loss was insignificant. The peritoneum was injured in one case and then the crevasse was clipped using Hem-o-Lock clips. There was no conversion to open procedures. The mean postoperative hospital stay was 5.3 days (range 4–6 days). The postoperative pathology indicated three cases of grade G1 pNEN and one case of grade G2 pNEN without subsequent therapy. The mean tumor size was 1.38 cm (range 1.0–1.8 cm) in diameter. All patients’ blood glucose level returned to normal range within 1 week postoperatively. Two patients had pancreatic biochemical leak [8], and their drainage tubes were removed in the tenth and seventeenth postoperative day, respectively. No patients underwent subsequent treatment, and no recurrence occurred during the 12-month follow-up period. The incisions in the lateral abdominal wall healed well, and the cosmetic results were satisfied by all patients.

## Discussion

Retroperitoneoscopic surgery was first applied and reported by urologist Bartel [9] and Gill [10]. After decades of development, mature surgical techniques have been established for retroperitoneoscopic surgery in the field of urology [11–13]. Nevertheless, because of the differences in surgeons’ habits and patients’ physiques, Asian doctors prefer retroperitoneoscopic surgery and doctors in western countries seem to be in favor of laparoscopic surgery. The application of robotic surgical system has further promoted the development of retroperitoneoscopic urological surgery [14].

We first completed and reported the retroperitoneoscopy in pancreatic surgery [1] and took the lead in accomplishing retroperitoneoscopic pancreatic enucleation [1, 2, 15], retroperitoneoscopic distal pancreatectomy [1, 2], and retroperitoneoscopic debridement for infected necrotizing pancreatitis [16]. Our experience in the dozens of operations indicates that the retroperitoneoscopic approach is safe and feasible for distal pancreatectomy and nucleation in selected patients, and has potential advantages over traditional laparoscopic approach [2]. For the treatment of infected necrotizing pancreatitis, retroperitoneoscopic approach could debride the necrotic tissue safely, effectively, and anatomically in single stage [16, 17]. The retroperitoneoscopic debridement has gradually gained popularity among surgeons in China.

Because of the limitation of operation space and disturbance of the kidney, RPS has limited operative extent and angle, which may compromise the operative safety to some extent. Although the application of robotic surgical system increases the preparation time and the number of ports, the intraoperative manipulation and the operative accuracy were significantly improved, as well as the operative safety and efficiency. In this study, four cases of RRPS were successfully completed, and the robotic system demonstrated that it is gentle, stable, accurate, and safe in intraoperative manipulation. But in terms of the enucleation, the advantage of RRPS is not significant. Only when precise procedures are involved in operation, the RRPS shows its advantage remarkably. The distal pancreatectomy requires precise dissection and separation of the distal pancreas form splenic vessels, which is difficult by RPS. Apart from the docking time of the robotic system, the operative time for distal pancreatectomy by RRPS seems to be shorter than that by RPS. Because of the awkward operative angle, suturing the pancreatic stump or splenic vessels by RPS is difficult. However, robotic instruments with 7° of freedom facilitate the suture, which remarkably improves the safety of operation and decreases the intraoperative blood loss. Three of the four patients had insignificant blood loss (no. 1, no. 3, and no. 4). The no. 3 patient had morbid obesity with BMI of 36.3 kg/m^2^, for which the distal pancreas is extremely hard to expose by traditional laparoscopic surgery. Nevertheless, the distal pancreas of this obese patient could be rapidly exposed and precisely detached from splenic vessels by RRPS. This operation approach is safe and efficient and with no intra-abdominal adhesion formation, which demonstrates the perfect combination of robotic surgical system and retroperitoneoscopy.

The unique advantages of robotic surgical system also could change part of the operating habits of surgeons for RPS. In RRPS, the splenic vessels could be easily dissected and mobilized. To separate the distal pancreas of the no. 2 patient, the splenic vessels were first ligated segmentally instead of being transected and then the distal pancreas was resected. This technique avoided resection of splenic vessels during distal pancreatectomy, thus modifying the Warshaw’s approach and decreasing the risk of peritoneum injury [18]. As the laparoscopic linear stapler is inconvenient in the retroperitoneal space in RRPS, the pancreas was suitable for transection by ultrasonic scalpel or electric hook and then the pancreatic stump was oversewed in a safe and efficient way by a robotic needle holder.

Same as RPS, RRPS is still not suitable for patients with malignant tumors and large volume lesions. When the peritoneum is severely injured in the operation, the operative space will be compressed and the instruments and vision confined, even with the help from the assistant port. Therefore, in order to avoid injury to peritoneum in RRPS, indications for RRPS should be strictly selected, great care be taken during operation, and anatomic landmark and surgical approach be clearly identified.

## Conclusion

Our preliminary clinical application of RRPS indicates that, for lesions in dorsal portion of distal pancreas, the safety of operation could be improved by RRPS, which is attributed to the straightforward exposure of the operative field as well as the steady vision and flexible instruments that are convenient for suture. However, the advantage of RRPS over RPS in enucleation is not significant. When precise procedures are involved in operation such as distal pancreatectomy, the RRPS displays its remarkable advantages. As this is a preliminary experience of RRPS, further clinical application and comparison studies are required to evaluate its significance.
